# Nogo-B receptor is required for stabilizing TGF-β type I receptor and promotes the TGF-β1-induced epithelial-to-mesenchymal transition of non-small cell lung cancer

**DOI:** 10.7150/jca.50483

**Published:** 2021-01-01

**Authors:** Donghua Wu, Baofeng Zhao, Yang Song, Xinming Chi, Hailu Fu, Tiantong Guan, Liyuan Zhang, Xueguang Yang, Ke Hu, Rong Huang, Xiaomeng Jin, Qing Robert Miao, Shujuan Shao

**Affiliations:** 1 Key Laboratory of Proteomics of Liaoning Province, Dalian Medical University, Dalian 116044, China.; 2 CAS Key Laboratory of Separation Sciences for Analytical Chemistry, National Chromatographic R&A Center, Dalian Institute of Chemical Physics, Chinese. Academy of Sciences, Dalian 116023, China.; 3 Division of Pediatric Surgery, Department of Surgery, Children's Research Institute, Medical College of Wisconsin, Milwaukee, WI 53226, USA.; 4 Divisions of Pediatric Pathology, Department of Pathology, Children's Research Institute, Medical College of Wisconsin, Milwaukee, WI 53226, USA.; 5 Department of Pharmacology and Toxicology, Medical College of Wisconsin, Milwaukee, WI 53226, USA.; 6 Foundations of Medicine, New York University Long Island School of Medicine, Mineola, NY 11501, USA.

**Keywords:** Non-small cell lung cancer, Nogo-B receptor, Metastasis, Epithelial-mesenchymal transition, Transforming growth factor-β signal pathway

## Abstract

**Background and Objective:** Metastasis is the leading cause of death in patients with advanced non-small cell lung cancer (NSCLC), and epithelial-mesenchymal transition (EMT) is a crucial event in the metastasis of NSCLC. Our previous works demonstrated that NgBR promoted EMT in NSCLC. However, the molecular mechanism was unclear.

**Methods:** TGF-β1 was used to induce EMT process of NSCLC cells. The biological functions of NgBR in promoting TGF-β1-induced NSCLC metastasis were studied by gain- and loss-of-function assays both *in vitro* and *in vivo*. The underlying mechanisms were studied using molecular biology assays.

**Results:** We found that knockdown of NgBR inhibited TGF-β1-induced cell migration and invasion in NSCLC cells. In contrast, NgBR overexpression promoted TGF-β1-induced EMT of A549 cells. Mechanically, we found that knockdown of NgBR facilitated ubiquitination and degradation of TβRI, leading to downregulation of TβRI expression in NSCLC cells. Moreover, we confirmed a positive correlation between NgBR and TβRI in NSCLC tissues.

**Conclusions:** Our findings provide a novel role of NgBR in modulating TGF-β1-induced EMT and propose NgBR as a new therapeutic target for treating NSCLC patients.

## Introduction

Lung cancer is one of the most severe diseases that endanger human health, with a high incidence and low survival rate 1-4. Among them, non-small cell lung cancer (NSCLC) accounts for about 85% of lung cancer and is divided into large cell lung cancer, lung squamous cell carcinoma and lung adenocarcinoma 5. The five-year survival rate of NSCLC is as low as 18%, which is caused by the difficulty of the diagnosis towards the early lung cancer, with the low diagnostic rate of 16% in an early stage 4, 5. Although the prevention, diagnosis, and treatment of lung cancer have been greatly improved in recent years, the survival rate of lung cancer patients still remains low 1, 3, 4, 6. Therefore, it is critical to clarify the molecular mechanism of the development of NSCLC further, to find a new target for more effective diagnosis and treatment, to improve the survival rate of NSCLC patients and improve the prognosis.

Lung cancer metastasis is the leading cause of death in patients with advanced NSCLC 7, and epithelial-mesenchymal transition (EMT) is a crucial event in the metastasis of NSCLC. In the EMT process, epithelial cells lose their original polarity and change into mesenchymal cells with enhancing ability of migration and invasion, simultaneously with the morphology changes from close connection and regular arrangement into loose connection and irregular arrangement 7-11. Down-regulation of E-cadherin is thought to be a hallmark of EMT 8, 12. At the same time, mesenchymal phenotypic biomarkers, such as N-cadherin, Vimentin, Snail, Twist, were upregulated 7, 8, 10, 13. Transforming growth factor (TGF)-β signal pathway plays an essential role in regulating EMT 11, 14. In this process, TGF-β1 first binds directly to TGF-β type II receptor (TβRII) to form a complex and phosphorylates TβRII. At this time, the conformation of TGF-β1 changes, which is recognized and combined by TGF-β type I receptor (TβRI) to form a TβRII-TGF-β1-TβRI protein complex, resulting in phosphorylation of TβRI. Then activated TβRI phosphorylates Smad2 and Smad3, promoting phosphorylated Smad2, Smad3 to form complexes with Smad4 in the cytoplasm, which translocate to the nucleus and regulate the transcription of EMT related genes 14, 15. In addition to activating the Smads signaling pathway, TGF-β receptors also activate downstream non-Smads signaling pathways, like ERK, PI3K-Akt, c-Jun N-terminal kinase (JNK), and p38 MAPK signaling pathways^11^, ^16^, ^17^, to promote NSCLC cell migration, invasion, and metastasis through the induction of EMT.

Nogo-B receptor (NgBR) is a receptor that specifically binds to Nogo-B 18. It is a single transmembrane protein that functions mainly in the remodeling and the formation of blood vessels 19-21. Studies in recent years have shown that NgBR plays an essential role in tumor progression 22, 23. Our previous research proved that in NSCLC, NgBR activates the downstream MEK/ERK signaling pathway by promoting Ras membrane localization and activation. The phosphorylated ERK1/2 (p-ERK1/2) enters the nucleus and binds to the promoter of Snail1 to promote its transcription, which in turn promotes EMT 24. However, it is still unknown whether NgBR also plays a crucial role in the TGF-β signaling pathway and involves in TGF-β1-mediated EMT.

Herein, we revealed that NgBR acted as a regulator of TGF-β1-induced EMT in NSCLC cells. Our study proposes a new perspective that NgBR regulates TβRI protein stability, which in turn activates TβRI downstream signaling pathways and promotes the ability of cell invasion and migration.

## Materials and Methods

### Cell lines and culture

Human NSCLC cell lines A549, H1299 were purchased from the American Type Culture Collection (Manassas, VA, USA), which were authenticated by STR profiling before distribution. The cell lines were cultured as described previously 24. The cells lines were treated with or without 5 ng/ml TGF-β1 for the indicated time, which was purchased from Invitrogen (Carlsbad, CA).

### Knockdown and overexpression of NgBR in NSCLC cells

A549 and H1299 cell lines were transient transfection with All-Star non-silencing siRNA (NS), NgBRsiRNA (siNgBR) and stably transfected with pIRES-NgBR plasmid (NgBR), pIRES-NC plasmid (NC), NgBRshRNA (shNgBR), Negtive control shRNA (shNC), all of these sequences were described previously 24 and selected with puromycin.

### Western blot analysis

Western blotting was performed as described previously 24. Primary antibodies included anti-E-cadherin, Vimentin, Snail1, Twist1, ubiquitin, β-actin antibodies (ProteinTech, Wuhan, China), phospho-Akt (Ser473), total Akt, p-ERK1/2 (Thr202/Tyr204), ERK1/2, phospho-Smad2 (Ser465/467), phospho-Smad3 (Ser423/425), Smad2, Smad3, Na^+^ and K^+^-ATPase antibodies (Cell Signaling Technology, Danvers, MA, USA), anti-TβRI antibody, anti-TβRII antibody, NgBR(Abcam, Cambridge, USA).

### Tumor cell migration and invasion assays

Transwell assay was carried out as described previously 24. 24-well-Transwells (Costar/Sigma, St Louis, MO, USA) were used without or with Matrigel (BD Biosciences, San Jose, CA, USA) coatings for migration and invasion assays, respectively. 50000 cells in 0.2 ml serum free RPMI-1640 supplement with TGF-β1 (5 ng/ml) were seeded into the upper chamber, and the lower chamber was filled with 0.5ml RPMI 1640 containing 10% FBS. After incubated for 12-24 h at 37 ºC, the chambers were washed with PBS, fixed with 4% paraformaldehyde for 15 minutes at room temperature, and stained with Crystal Violet solution for 10 minutes. Cells on the upper surface of the filter were wiped off gently, while cells migrated or invaded into the bottom surface were then counted under an inverted light microscope (Olympus, Japan).

### Immunofluorescence staining

After A549 cells were treated with TGF-β1 (5 ng/ml) for the indicated time, immunofluorescence staining was performed as described previously 24. Cells were seeded on the cover slips with the density at 50%-70% confluency. Then, the cells were washed with PBS and fixed on 4% paraformaldehyde for 15 minutes at room temperature, with or without permeabilizing by 0.3% triton, blocking with 5% goat serum at 37°C for 30 minutes. These cover slips were incubated with the specified primary antibody diluted in PBS overnight at 4°C in the wet box. In the next day, cells were incubated with fluorescence secondary antibody at 37°C for 1 h, washed by PBS and stained with DAPI for 5 minutes.

### Immunohistochemistry

NSCLC tissue microarrays (TMA) including 30 positive lymph nodes of NSCLC patient were purchased from Shanghai Outdo Biotech (Shanghai, China). The study was approved by the Ethics Committee of Dalian Medical University. Immunohistochemistry (IHC) was carried out as described previously 24. IHC was used to assess the expression of NgBR, TβRI and E-cadherin. Before the antigen retrieval with citrate buffer (PH 6.0) in a microwave oven, the paraffin-embedded tissue arrays were baked at 70°C, dewaxed in xylene andrehydrated in ethanol at the specified time. After quenching endogenous peroxides with 3% hydrogen peroxide (H_2_O_2_) at room temperature, the TMA were blocked with 5% goat serum at room temperature for 20 minutes, and then incubated with the respective NgBR, TβRI or E-cadherin antibodies diluted in PBS overnight at 4°C in the wet box. In the next day, the TMA were incubated in pv-9000 kit and color development was performed using 3,3-diaminobenzidin (DAB) (ZSGB-Bio, Beijing, China). Afterwards, the TMA were counterstained using hematoxylin and dehydrated through increasing concentrations of ethanol and xylene.

### Cycloheximide (CHX) assays

Cells were treated with 100 mg/ml CHX (Sigma, USA) for 0 h, 0.5 h, 1 h, 2 h, 3 h and 5 h to block protein synthesis. The protein levels of TβRI were assessed by Western blot analysis.

### Ubiquitination assays and immunoprecipitation

Cells were treated with 10 μm MG132 (SELLECK, USA) for 6 h, and then the whole-cell lysates were extracted for immunoprecipitation (IP) to examine protein ubiquitination. The IP assay was carried out with the Pierce Co-IP Kit (Thermo Scientic) according to the manufacturer's protocol 23. 8 μg of the affinity-purified ubiquitin or TβRI antibody were coupled with the resin at room temperature for 2 h. 500 μg of whole-cell lysates were mixed with the resin and the prepared appropriate experimental controls, incubating with gentle mixing for overnight at 4°C. The eluted complex samples were analyzed by Western blot.

### Animal experiments

The IACUC protocol was approved by the Animal Care and Ethics Committee of Dalian Medical University. Ten million of A549 cells either stably overexpressing NgBR or control vector (NC) suspended in 100 μl normal saline were injected into the right side of axillary subcutaneous of male BALB/c nude mice (4 weeks old), which were purchased from Charles River Laboratories (Beijing, China) and bred in a specific-pathogen-free environment in an animal facility of Dalian Medical University. For each group of 5 mice, the size of the tumor was measured with a vernier caliperevery 4 days. After the observation for 1 month, the nude mice were euthanized to collect tumor and lung tissues. The morphology of tumor cells was examined by H&E staining and protein expressions of E-cadherin, TβRI, NgBR protein were analyzed by immunohistochemistry.

### Statistical analysis

All statistical analyses were carried out by SPSS 23.0 (SPSS, Chicago, IL, USA) or GraphPad Prism 7.0 software (GraphPad Software, La Jolla, CA, USA). All experiments were repeated independently at least three times and data were expressed as mean ± SD. A two-tailed paired *t*-test was analyzed for two preselected groups, while *p* < 0.05 was considered as statistically significant.

## Results

### NgBR promotes TGF-β1-induced cell migration and invasion

**Figure 1:**
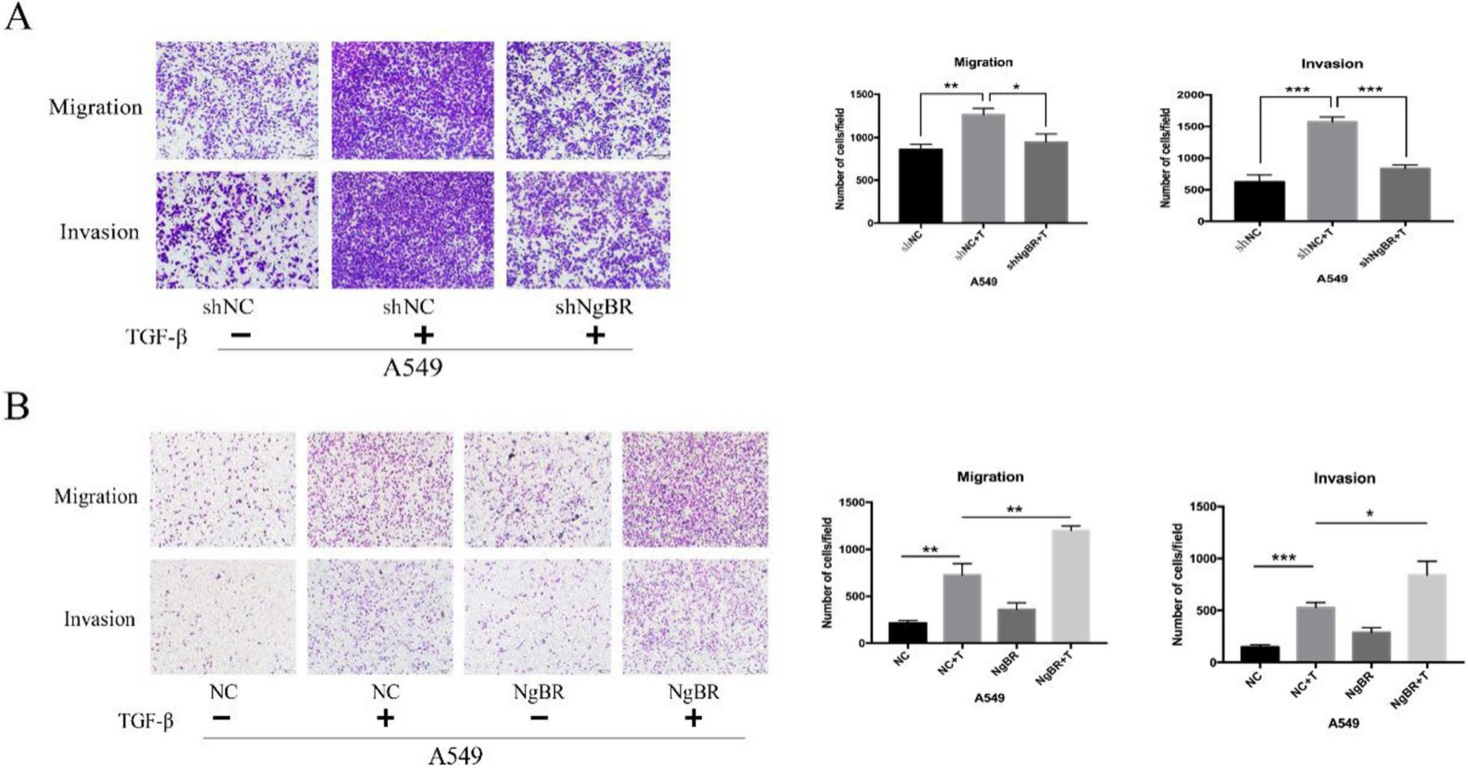
** NgBR is required for TGF-β1-induced cell migration and invasion of A549 cells.** A, Transwell assays were used to assess the migration and invasion capacity of stably NgBR knockdown A549 cells with TGF-β1 treatment (5 ng/ml). Scale bar, 100 µm. Error bar, SD of three independent experiments. *p<0.05, **p<0.01 and ***p<0.001. B, Transwell assays were used to assess the migration and invasion capability of NgBR overexpressed A549 cells with or without TGF-β1 treatment (5 ng/ml). Scale bar, 100 µm. Error bar, SD of three independent experiments.*p<0.05, **p<0.01 and ***p<0.001.

We analyzed the effect of NgBR on TGF-β1-mediated cell migration and invasion. Results of transwell assays showed that NgBR knockdown significantly inhibited TGF-β1-induced migration and invasion of both A549 and H1299 cells (Fig. 1A and S1). Conversely, NgBR overexpression obviously enhanced TGF-β1-induced migration and invasion of A549 cells (Fig. 1B). These results show that NgBR promotes TGF-β1-induced cell migration and invasion.

### NgBR promotes TGF-β1-induced EMT

**Figure 2:**
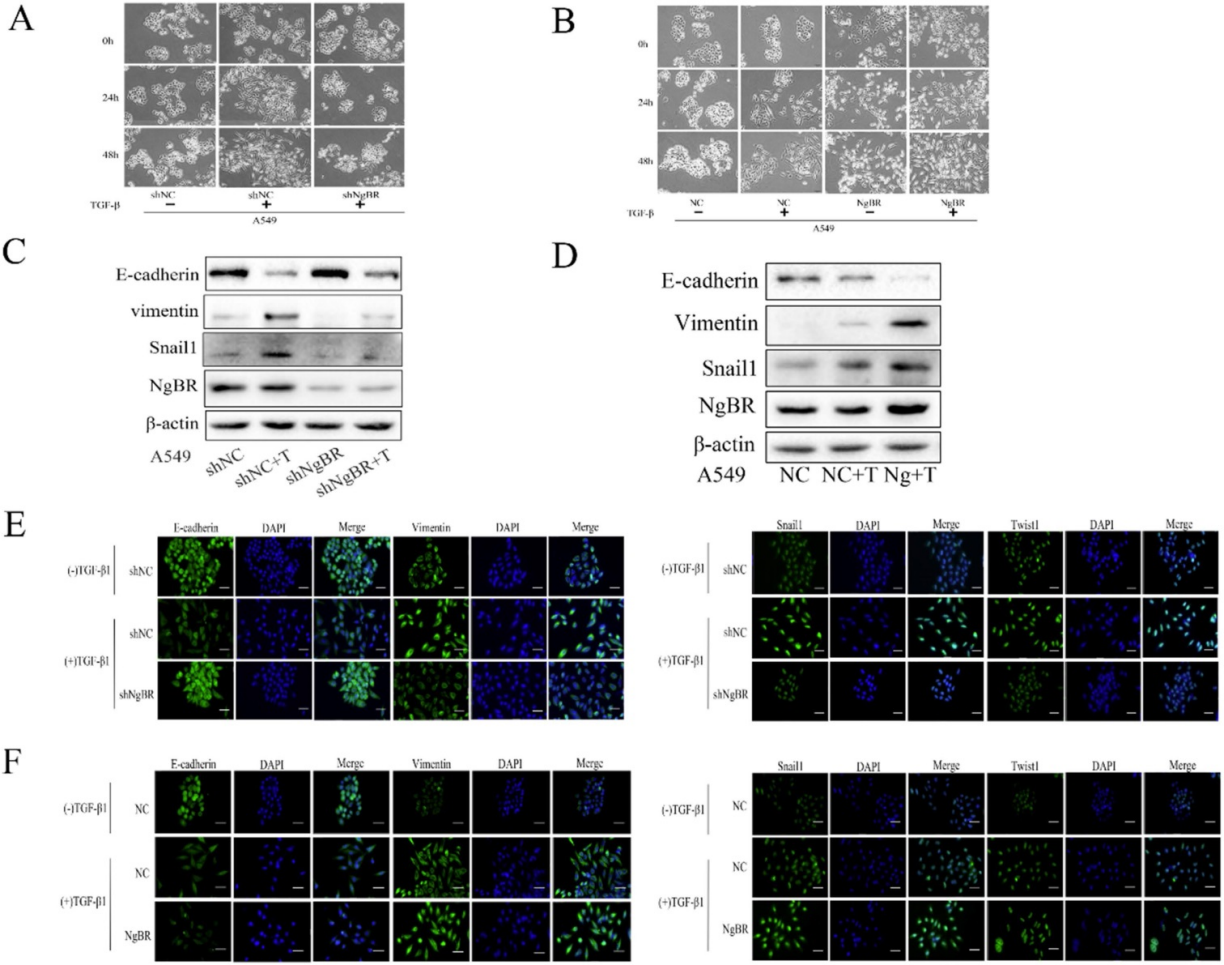
** NgBR is required for TGF-β1-induced EMT process in A549 cells.** A, cells morphology in NgBR knockdown A549 cells with or without TGF-β1 treatment (5 ng/ml) for indicated time. Scale bar, 200μm. B, cells morphology in NgBR overexpressed A549 cells with or without TGF-β1 treatment (5 ng/ml) for indicated time. Scale bar, 200 µm. C and D, E-cadherin, Vimentin and Snail1 expression levels were detected by using Western blot analysis in NgBR knockdown (C) or overexpressed (D) A549 cells with or without TGF-β1 treatment (5 ng/ml).β-actin was used as a housekeeping control. E, A549 cells stably transfected with NgBR shRNA (shNgBR) or nonspecific control (shNC) were treated with TGF-β1 (5 ng/ml) for 48h and then subjected to immunostaining with E-cadherin, Vimentin, Snail1 and Twist1 antibodies. While cell nuclei were stained with DAPI. Scale bar, 37 µm. F, A549 cells stably transfected with pIRES-NC (NC) or pIRES-NgBR (NgBR) were treated with TGF-β1 (5 ng/ml) for 48 h and then subjected to immunostaining with E-cadherin, Vimentin, Snail1 and Twist1 antibodies. While cell nuclei were stained with DAPI. Scale bar, 37 µm.

To explore the underlying mechanism by which NgBR promotes TGF-β1-induced cell migration and invasion, we analyzed the effects of NgBR on TGF-β1-induced EMT of A549 cells. We first observed cell morphology changes of A549 cells. As shown in Fig. 2A, A549 cells treated with TGF-β1 began to turn into a spindle-like and elongated cell phenotype at 24 h, which was more pronounced at 48 h. Interestingly, the TGF-β1-induced cell morphology changes were diminished significantly in NgBR knockdown A549 cells. In contrast, NgBR overexpression in A549 cells dramatically enhanced TGF-β1-induced cell morphology changes, showing more elongated and more dispersed cells (Fig. 2B). We further detected the levels of TGF-β1-induced EMT marker proteins in A549 cells by Western blot and immunofluorescence staining. We found NgBR knockdown attenuated TGF-β1-induced upregulation of Vimentin, Snail1 and Twist1, as well as downregulation of E-cadherin (Fig. 2C, 2E, and S2). However, overexpression of NgBR enhanced the TGF-β1-induced changes of these EMT marker proteins (Fig. 2D and 2F). Thus, these results indicate that NgBR increases TGF-β1-induced cell migration and invasion by promoting TGF-β1-induced EMT process.

### NgBR enhanced TGF-β1-stimulated EMT signaling pathway

**Figure 3:**
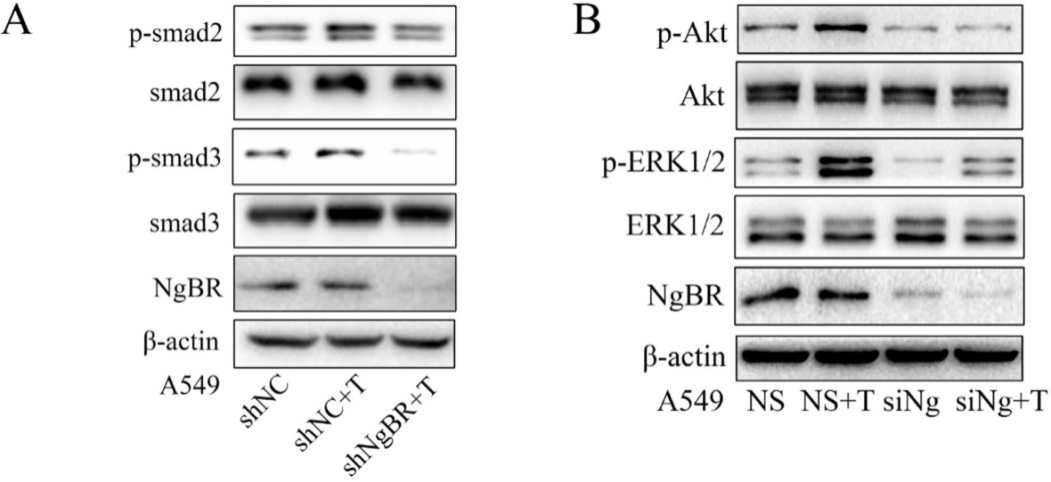
** NgBR promotes TGF-β1-stimulated both Smad and non-Smad pathway.** A, A549 cells stably transfected with NgBR shRNA (shNgBR) or nonspecific control (shNC) were treated with TGF-β1 (5 ng/ml) for 1h and then subjected to Western blot analysis. B, A549 cells transfected with NgBR siRNA (siNg) or All-Star non-silencing siRNA (NS) were treated with TGF-β1 (5 ng/ml) for 1h and then extracted whole-cell lysate for Western blot analysis.

Previous studies showed that TGF-β1 promotes EMT by both Smad and non-Smad-dependent pathways 11, 25-27. To determine the molecular mechanism by which NgBR promotes TGF-β1-induced EMT process, we examined the effects of NgBR on TGF-β1-induced both Smad and non-Smad signaling pathways. The Western blot results showed that NgBR knockdown abolished the TGF-β1-induced phosphorylation of Smad2 and Smad3 (p-Smad2 and p-Smad3) (Fig. 3A). In contrast, TGF-β1-stimulated phosphorylation of Smad2 and Smad3 were enhanced in A549 cells overexpressing NgBR (Fig. S3A). The similar change pattern was also observed on the TGF-β1-stimulated phosphorylation of AKT and ERK1/2 in NgBR knockdown and overexpressed A549 cells (Fig. 3B and S3B). Furthermore, we detected the nuclear localization of Smad2/3 by using immunofluorescence staining and results indicated that knockdown of NgBR blocked TGF-β1-induced Smad2/3 translocation into the nucleus (Fig. S3C). These findings indicate that NgBR promotes TGF-β1-induced EMT process by enhancing Smad dependent and independent TGF-β1 signaling pathways.

### NgBR increases TβRI expression in NSCLC cells

**Figure 4:**
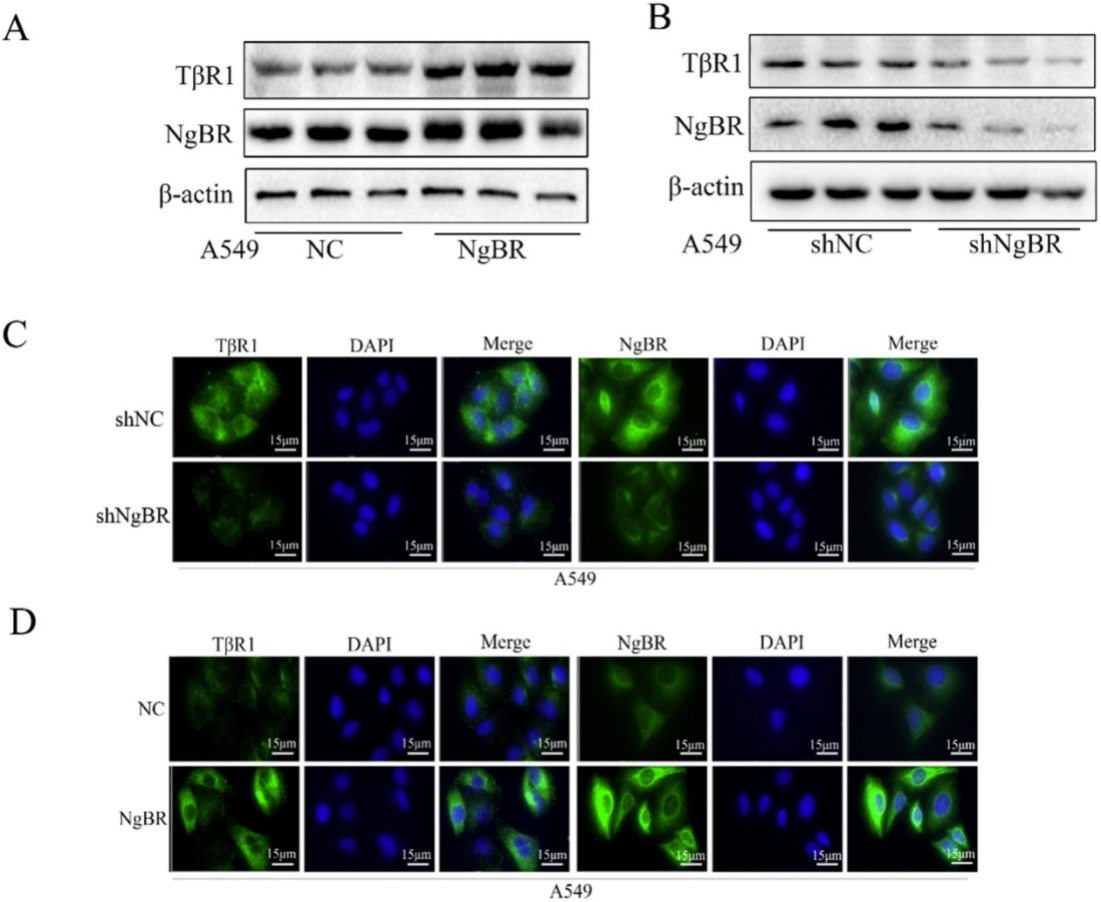
** NgBR prevents TβRI degradation in A549 cells.** A, expression of TβRI and NgBR were examined by using Western blotting in NgBR overexpression A549 cells. β-actin was used as a loading control. B, expression of TβRI and NgBR were examined by using Western blotting in NgBR knockdown A549 cells. β-actin was used as a loading control. C and D, A549 stably knockdown NgBR (C) and overexpression NgBR cells (D) were immunostained with the TβRI and NgBR antibody, while cell nuclei were stained with DAPI staining. Scale bar, 37 µm.

Suggesting NgBR may play a crucial role in the upstream of the TGF-β signaling pathway since NgBR is required for TGF-β1-induced Smad and non-Smad signaling pathways, we examined the effects of NgBR on TβRI and TβRII expression. Western blot and immunofluorescence staining results revealed the protein levels of TβRI was increased in A549 cells overexpressing NgBR and decreased in NgBR knockdown A549 cells (Fig. 4A-D). The same regulatory effects of NgBR on the protein levels of TβRI were also observed in H1299 cells (Fig. S4A and S4B). However, there were no significant changes in protein levels of TβRII in both A549 and H1299 cells either overexpressing NgBR or knocking down NgBR (Fig. S4C and S4D). These results suggest that NgBR enhance TGF-β1-stimulated signaling pathway by increasing the protein levels of TβRI but not TβRII in lung cancer cells.

### NgBR interacts with TβRI and blocks its degradation

**Figure 5:**
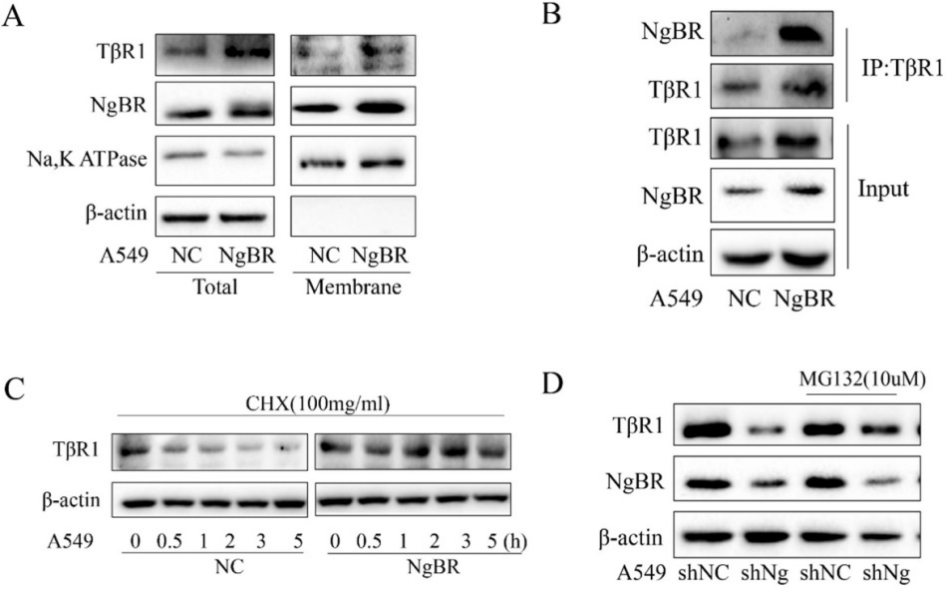
** NgBR enhances TβRI stability in A549 cells.** A, The whole-cell lysates and cell membrane protein were extracted from A549 cells stably transfected with pIRES-NC (NC) or pIRES-NgBR (NgBR) to evaluate the expression of TβRI. Na, K ATPase and β-actin were used as a housekeeping control for the membrane and total lysates, respectively. B, Whole-cell lysates of A549 cells were immunoprecipitated with TβRI antibody, and then protein levels of TβRI and NgBR were detected by Western blot. C, A549 cells stably transfected with pIRES-NC (NC) or pIRES-NgBR (NgBR) were treated with CHX for 0 h, 0.5 h, 1 h, 2 h, 3 h and 5 h, and then the whole-cell lysates were extracted for Western blot analysis. D, A549 cells stably transfected with NgBR shRNA (shNgBR) or nonspecific control (shNC) were treated with or without MG132 (10 µM) for 6 h, then the whole-cell lysates were extracted for Western blot analysis.

NgBR is a membrane protein that is the gateway for signaling transduction 18, 28. Previous research proposed that degradation of TβRI serves as an important regulatory mechanism for TGF-β signaling 29-33. Thus, we further tested the hypothesis that NgBR may interact with TβRI on the cell membrane and promote the protein stability of TβRI. Our Western blot results showed that NgBR overexpression increased TβRI protein levels in both total cell lysates and membrane fractions of A549 cells (Fig. 5A); whereas NgBR knockdown decreased TβRI protein levels in both total protein and membrane fraction of H1299 cells (Fig. S5A). The results of TβRI immunoprecipitation demonstrated that NgBR interacted with TβRI and NgBR overexpression significantly increased the association of NgBR with TβRI in A549 cells (Fig. 5B). Conversely, NgBR knockdown by shRNA decreased the interaction of NgBR with TβRI in H1299 cells (Fig. S5B). Previous reports 34 showed that TβRI protein undergone degradation via the ubiquitin proteasome pathway. To determine the role of NgBR in regulating ubiquitination and degradation of TβRI proteins in lung cancer cells, the degradation dynamics assay was carried out in A549 cells. We used cycloheximide to inhibit protein synthesis so that constant degradation of TβRI was appreciated by the decreased protein levels. Our data showed that NgBR overexpression led to a prolonged half-life of TβRI in A549 cells (Fig. 5C). To further confirm the NgBR-dependent degradation of TβRI protein, we treated A549 and H1299 cells with proteasome inhibitor MG132. Consistently, the degradation of TβRI caused by NgBR knockdown was blocked by MG132 in A549 and H1299 cells (Fig. 5D and S5C). The results of ubiquitin immunoprecipitation further showed that NgBR knockdown increased the ubiquitination of TβRI in H1299 cells (Fig. S5D). These results suggest that NgBR is required for maintaining substantial protein levels of TβRI by reducing the degradation of TβRI via ubiquitin proteasome pathway.

### NgBR expression correlates with TβRI *in vivo*

**Figure 6:**
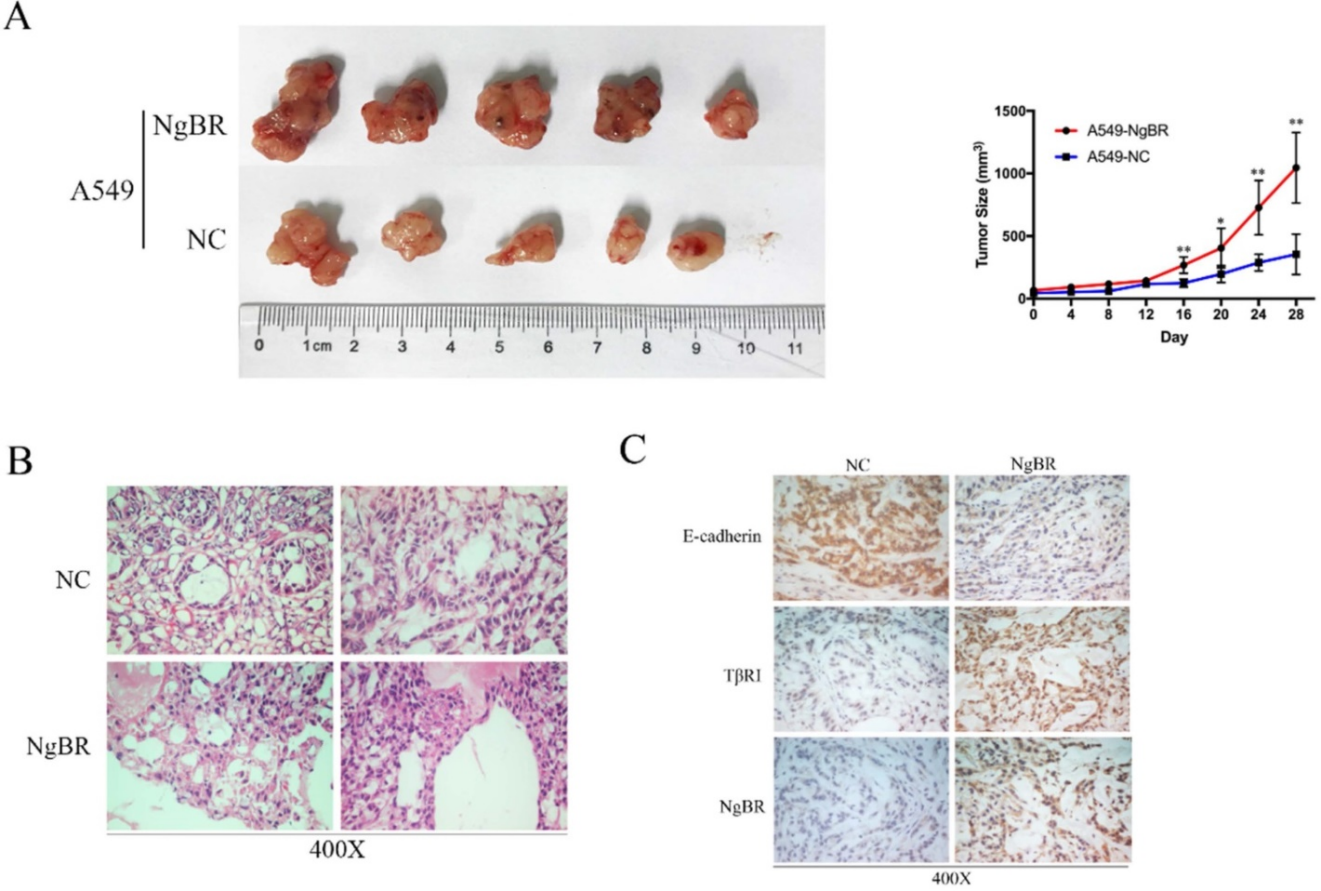
** NgBR expression correlates with TβRI* in vivo.***A, the representative images of A549-NC and A549-NgBR tumor xenografts (left panel). Statistical analysis of tumor volumes (right panel), Error bar, SD of three independent experiments. *p<0.05, **p<0.01. B, H&E staining of the A549-NgBR and A549-NC tumor xenografts. Scale bar, 20 µm. C, Representative images of E-cadherin, TβRI and NgBR immunohistochemical staining of the A549-NgBR and A549-NC tumor xenografts. Scale bar, 20 µm.

To further examine the correlation of NgBR and TβRI *in vivo*, we established tumor xenograft model by injecting A549 cells overexpressing NgBR (A549-NgBR), A549 cells overexpressing vector-only control (A549-NC). As shown in Fig. 6A, the growth of tumor was faster, which led to larger tumor volume in A549-NgBR tumor xenografts. H&E staining confirmed that there are many necroses in A549-NgBR tumor xenografts (Fig. 6B). IHC staining further showed that TβRI immunostaining intensity was significantly increased in A549-NgBR tumor xenografts as compared with A549-NC tumor sections (Fig. 6C). In contrast, the immunostaining intensity of E-cadherin, the epithelial cell marker, was decreased in A549-NgBR tumor xenografts (Fig. 6C). Furthermore, we examined the expression of TβRI and NgBR in metastasized tumors in lymph node tissues of lung adenocarcinoma patients. Consistently, NgBR and TβRI were highly expressed in lung cancer cells of lymph node metastasis (Fig. S6). These results demonstrate the positive correction of NgBR and TβRI *in vivo*.

## Discussion

Our previous studies showed that NgBR promotes EMT in NSCLC and breast cancer cells 24, 35. Here, we further demonstrated the role of NgBR in TGF-β1-mediated EMT of NSCLC. Our current data suggest that NgBR is required for the stability of TβRI but not TβRII. Overexpression of NgBR prevents the degradation of TβRI and enhances the TGF-β-stimulated signaling, which promotes the EMT of NSCLC cells. This is the first report to elucidate the crucial contribution of NgBR to TGF-β1-mediated signaling pathway and EMT process.

TGF-β signaling pathway is considered to play a key role in EMT process 11, 36, which is essential for the invasion and migration of cancer cells 36, 37. The major characteristic of EMT is the disintegration and disassembly of cell-cell junctions that leads cells to become spindle-like phenotype 36. Our results showed NgBR knockdown abolished the TGF-β1-induced cell phenotype changes and overexpression of NgBR enhanced the TGF-β1-induced cell phenotype changes, indicating NgBR is required for TGF-β1-mediated EMT process. In addition, NgBR dependent cell invasion and migration further illustrated the important role of NgBR in promoting the TGF-β signaling pathway.

TGF-β1-induced EMT is a complicated process. The change of molecular hallmarks is the downregulation of epithelial cell marker E-cadherin, accompanied by the upregulation of mesenchymal cell markers N-cadherin, Vimentin as well as EMT-related transcription factors Snail, Twist and Zeb 7, 25. Our previous study indicated NgBR was necessary for EMT of NSCLC cells and NgBR overexpression increased the expression of N-cadherin and Vimentin and decreased E-cadherin expression 24. Results of the present study further showed that overexpression of NgBR promoted the TGF-β1-induced EMT process and knockdown of NgBR reduced the TGF-β1 induced EMT. All these results reveal NgBR enhances TGF-β1-induced cell migration and invasion by promoting TGF-β1-induced EMT process.

The canonical TGF-β signaling pathway is that phosphorylated TβRI actives Smad2 and Smad3, and then phosphorylated Smad2 and Smad3 form a complex with Smad4, which translocates into the nucleus where the Smads complex cooperates with DNA binding transcription factors to activate or repress the transcription of target genes 25, 38. TGF-β also has Smad-independent signaling pathways through the activation of the p38 and Jun N-terminal kinase (JNK) mitogen-activated protein kinase (MAPK) pathways 39, 40. We previously showed that NgBR promotes the activation of Ras-MEK/ERK pathway by increasing Ras plasma membrane translocation, and results in the upregulation of Snail1 expression 24. In the present study, we found that NgBR knockdown abrogated TGF-β-induced activation of Smad2 and Smad3, while NgBR overexpression enhanced the TGF-β-induced activation of Smad2 and Smad3. Consistently, NgBR is also required for TGF-β-induced non-Smad pathway. NgBR knockdown also abrogated TGF-β-induced activation of Akt and MAPK signaling, while NgBR overexpression enhanced the TGF-β-induced activation of TGF-β-induced activation of Akt and MAPK signaling. As a cell membrane protein 18, NgBR should be a critical player in upstream of the TGF-β signaling pathway.

TGF-β receptors are the gateway for the TGF-β signaling transduction. The stability of TGF-β receptors is an essential regulatory mechanism for TGF-β signaling 25. We found that NgBR overexpression increased the protein expression of TβRI, but not TβRII in NSCLC cell. Consistently, knockdown of NgBR reduced the protein expression of TβRI. We also further demonstrated that NgBR was positively correlated with TβRI in NSCLC tissues. Ubiquitination modification is a key way to affect the stability of TβRI 29. Previous studies have found that protein stability of TβRI is affected by Syntenin which inhibits caveolin-mediated TβRI internalization 34 and USP4 which acts as a deubiquitylating enzyme to control TβRI levels at the plasma membrane 41. In the present study, we found NgBR interacted with TβRI and knockdown of NgBR increased the degradation of TβRI by promoting its ubiquitination in NSCLC cells. Consistently, we further showed that NgBR overexpression led to a prolonged half-life of TβRI in NSCLC cells. All these results suggest that NgBR is required for stabilizing TβRI by reducing the degradation of TβRI via ubiquitin proteasome pathway.

In summary, the present study indicates NgBR has an important role in enhancing TGF-β1-induced EMT process and cell migration and invasion in NSCLC cells. In addition, NgBR could serve as a novel regulatory protein in the TβRI ubiquitination and degradation in NSCLC cells. Consequently, overexpression of NgBR could activate both the Smad and non-Smad pathway induced by TGF-β1. This study further provides sufficient evidence that NgBR can serve as a NSCLC patient's therapeutic target.

## Supplementary Material

Supplementary figures.
