# Supplementary material for: Nogo-B receptor is required for stabilizing TGF-β type I receptor and promotes the TGF-β1-induced epithelial-to-mesenchymal transition of non-small cell lung cancer
Source: J Cancer. 2021 Jan 1;12(3):717–25. doi: 10.7150/jca.50483 (PMC7778533; doi:10.7150/jca.50483)
Supplement: Supplementary file 1 — Supplementary figures. [file jcav12p0717s1.pdf]

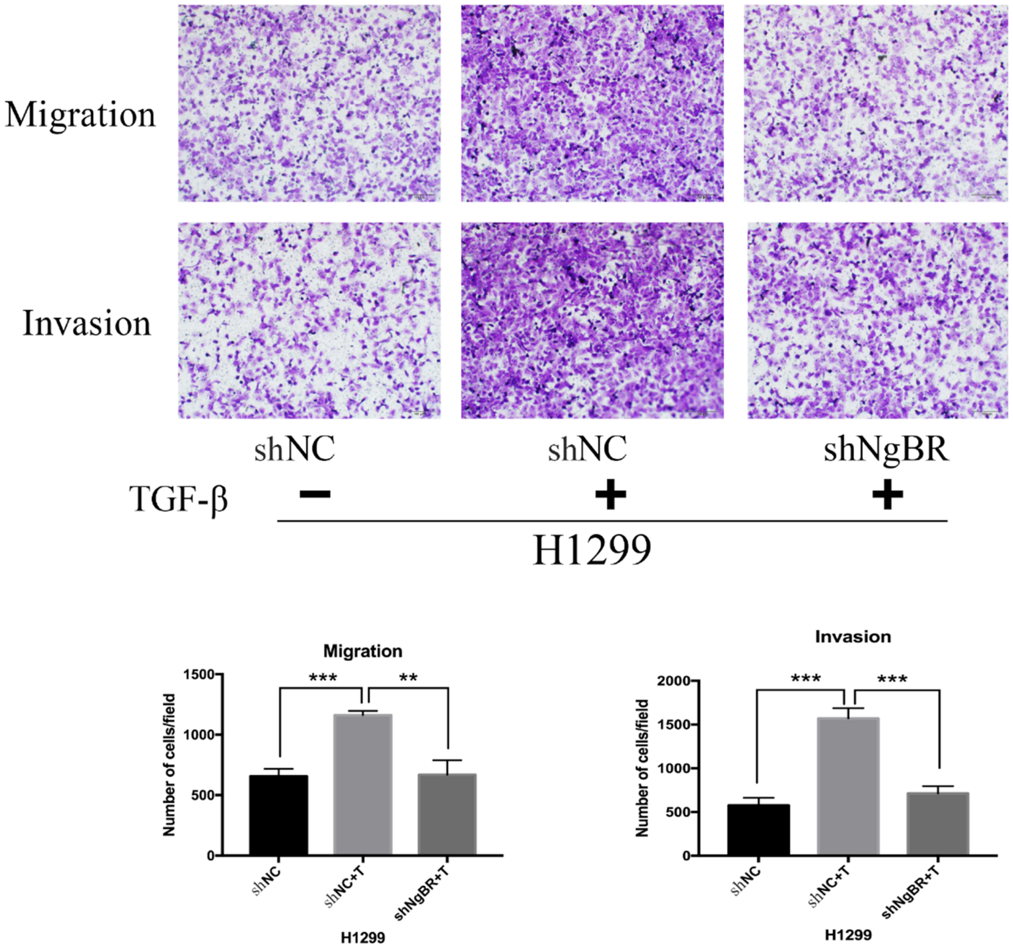

Fig. S1 NgBR is required for TGF-β1-induced cell migration and invasion in H1299 cells. Transwell assays were used to assess tumor cells migration and invasion capacity of NgBR knockdown H1299 cells with or without TGF-β1 treatment (5ng/ml). Scale bar, 100μm. Error bar, SD of three independent experiments. $p<0.01$** and $p<0.001$***.

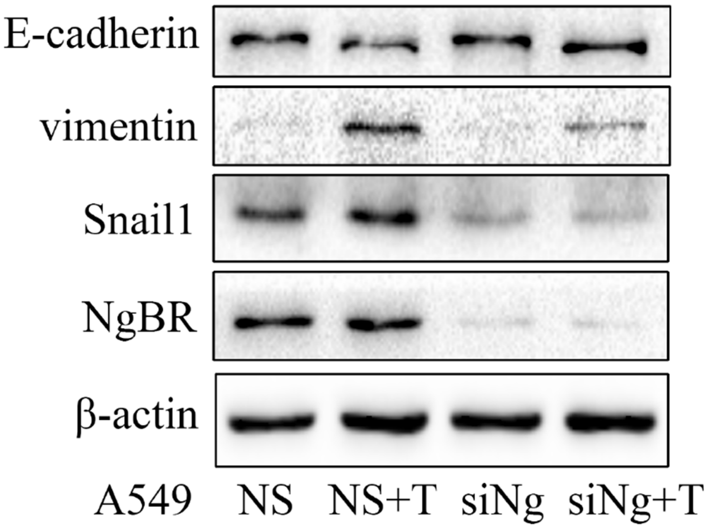

Fig. S2 NgBR knockdown inhibits TGF-β1-induced EMT process of A549 cells. A549 cells transfected with NgBR siRNA (siNg) or All-Star non-silencing siRNA (NS) were treated with or without TGF-β1 (5 ng/ml) for 48h and then extracted whole-cell lysates were used for Western blot analysis.

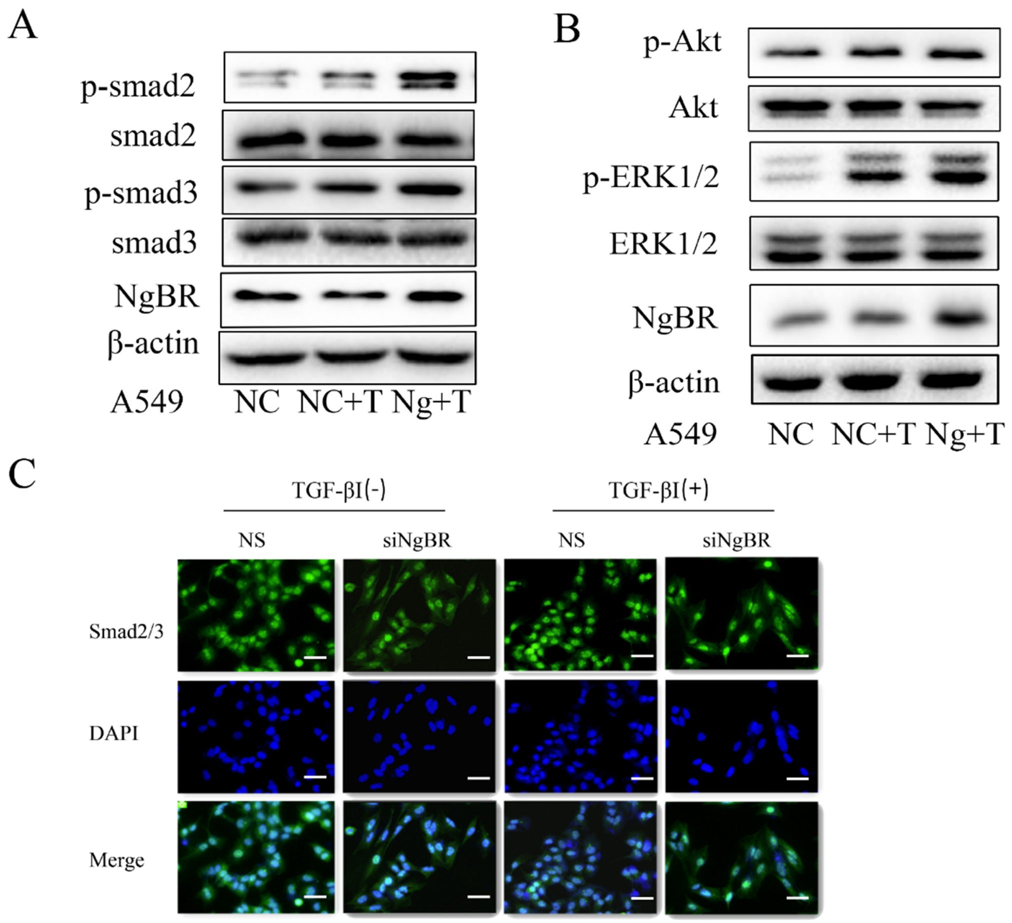

Fig. S3 NgBR knockdown blocks TGF-β1-induced translocation of smad2/3 into the nucleus. A and B, A549 cells stable transfected with pIRES-NC (NC) or pIRES-NgBR (NgBR) were treated with TGF-β1 (5 ng/ml) for 1h and then subjected to Western blot analysis. C, A549 cells transfected with NgBR siRNA (siNg) or All-Star non-silencing siRNA (NS) were treated with TGF-β1 (5 ng/ml) for 1h and then were subjected to immunostaining with smad2/3 antibodies. While cell nuclei were stained with DAPI. Scale bar, 37 μm.

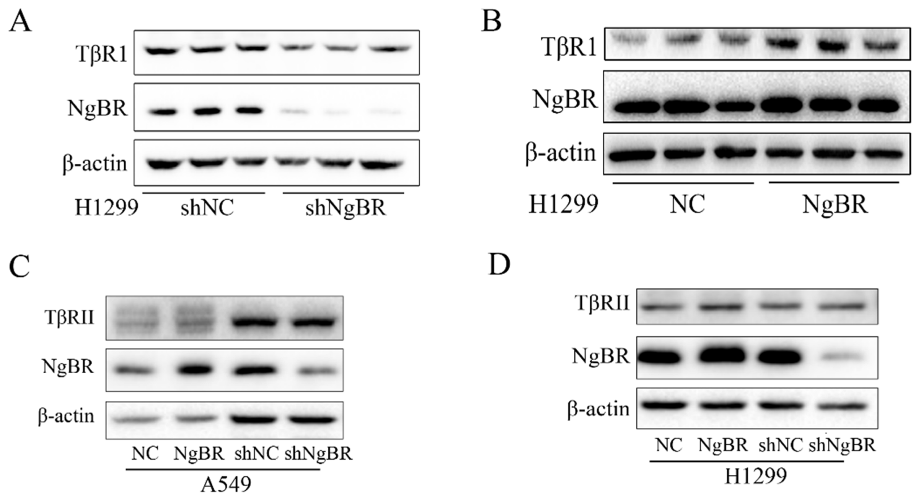

Fig. S4 NgBR is required for the stability of TβRI in H1299 cells. A and B, protein levels of TβRI and NgBR were examined by using Western blotting in NgBR stably knockdown NgBR (A) or stably overexpression (B) H1299 cells. C, protein levels of TβRII and NgBR in NgBR stable overexpression and NgBR stable knockdown A549 cells were examined by using Western blotting. D, protein levels of TβRII and NgBR in NgBR stable overexpression and NgBR stable knockdown H1299 cells were examined by using Western blotting. β-actin was used as a housekeeping control.

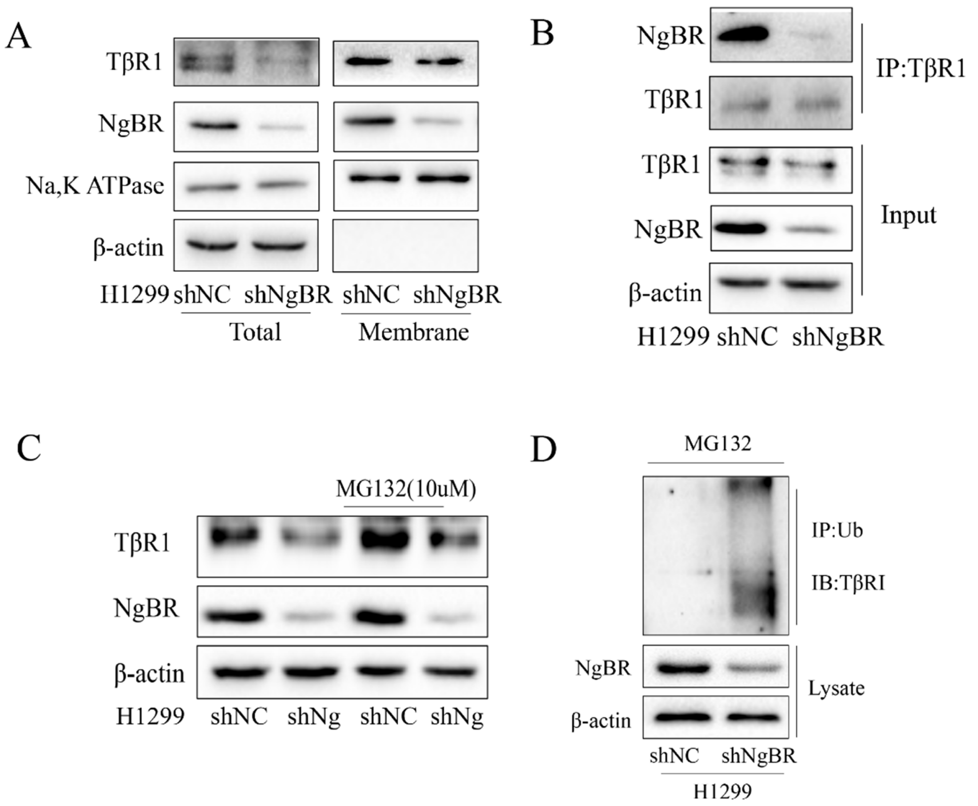

Fig.S5 NgBR prevents TβRI degradation in H1299 cells. A, the whole-cell lysates and cell membrane proteins were extracted from H1299 cells stably transfected with NgBR shRNA (shNgBR) or nonspecific control (shNC) to evaluate the alteration of NgBR and TβRI protein levels. Na, K ATPase and β-actin were used as housekeeping control for the membrane and total lysates, respectively. B, Whole-cell lysates of H1299 cells were immunoprecipitated with TβRI antibody, and then NgBR were detected by Western blot. C, H1299 cells stably transfected with NgBR shRNA (shNgBR) or nonspecific control (shNC) were treated with MG132 (10μM) for 6h, then the whole-cell lysates were extracted for Western blot analysis. D, H1299 cells stably infected with NgBR shRNA (shNgBR) or nonspecific control (shNC) were treated with MG132 (10μM) for 6h. Whole-cell lysates were immunoprecipitated with ubiquitin antibody and TβRI protein were detected by Western blot.

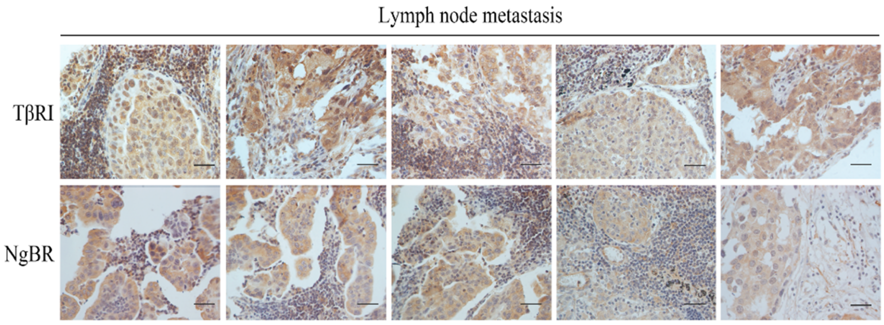

Fig.S6 Representative images of TβRI and NgBR immunohistochemical staining in lymph node metastasis in lung adenocarcinoma. Scale bar, 20 μm.
